# Surgical treatment of Bouveret Syndrome without completion cholecystectomy

**DOI:** 10.1016/j.ijscr.2025.111248

**Published:** 2025-04-02

**Authors:** Carrie Tackett, Patrick Stahl, Anthony McCloud, Joseph Eisner

**Affiliations:** Community Memorial Hospital, 147 Brent St., Ventura, CA 9300, United States of America

**Keywords:** Bouveret syndrome, Incomplete cholecystectomy, Case reports

## Abstract

**Introduction and importance:**

Bouveret syndrome, the rarest variant of gallstone ileus, occurs due to the passage of a gallstone into the gastrointestinal tract via a bilioenteric fistula, leading to gastric outlet obstruction. This condition represents less than 0.1 % of all mechanical bowel obstructions, predominantly affecting elderly females. Its nonspecific symptoms, including nausea, vomiting, and abdominal pain, often result in delayed diagnosis and significant mortality rates ranging from 12 % to 30 %. Historically, management involves a two-stage surgical approach: initial stone removal followed by interval cholecystectomy and fistula repair to prevent recurrence.

**Case presentation:**

This report presents two cases of younger patients with Bouveret syndrome managed with a single-stage surgical approach consisting of gallstone removal without subsequent cholecystectomy or fistula repair. Both patients remained symptom-free for two years postoperatively.

**Clinical discussion:**

These findings challenge the necessity of routine interval cholecystectomy and fistula closure, particularly in patients without recurrent biliary symptoms. We discuss the implications of a simplified surgical strategy, highlighting the potential for spontaneous fistula closure or adaptation of bile drainage pathways.

**Conclusion:**

While current literature supports interval surgery for younger patients to mitigate long-term risks, these cases suggest that selective management may reduce morbidity without compromising outcomes. Further research is needed to refine guidelines for the surgical management of Bouveret syndrome.

## Highlights

• Two patients with Bouveret Syndrome were treated with gallstone removal
• Neither patient had a fistula closure or cholecystectomy
• Two years post-operatively, both patients are doing well without recurrent symptoms
• Fistula closure and cholecystectomy have been described as the standard of care
• These two patients are evidence that may not be required

## 1 Introduction

Bouveret syndrome is a rare variant of gallstone ileus, in which a gallstone enters the gastrointestinal tract through a bilioenteric fistula, leading to mechanical obstruction. [1]. In the case of Bouveret syndrome, the gallstone becomes lodged in the duodenal bulb or proximal duodenum, causing gastric outlet obstruction. Bouveret syndrome constitutes approximately 1–3 % of all incidents of gallstone ileus, representing less than 0.1 % of all mechanical bowel obstructions [2]. The syndrome predominantly affects females and the elderly, with a female-to-male sex ratio of 1.86 and a mean age of 74 [3]. The symptoms are often nonspecific, typically manifesting as nausea, vomiting, or abdominal pain, which results in elevated mortality rates ranging from 12 % to 30 % [4].

Historically, surgical management has involved two-stage surgery for these patients [5]. The first stage is to remove the gallstone, relieving the gastric outlet obstruction, and the second stage is a cholecystectomy with fistula closure. It was previously thought that patients who did not undergo the second stage of surgery were prone to developing recurrent symptoms, especially in younger patients. However, we present two cases from a private practice setting of Bouveret Syndrome in younger patients with no recurrent symptoms after two years without the second-stage surgery.

## 2 Cases

### 2.1 Patient #1

A 60-year-old female presented to the emergency department with a 3-day history of nausea and vomiting. She was unable to tolerate any oral intake, but had no changes in bowel habits, endorsing normal flatus without constipation or diarrhea. Her medical history was notable for anxiety, diet-controlled type 2 diabetes mellitus, and a history of smoking, with occasional use of marijuana and alcohol a few times per month.

**Fig. 1:**
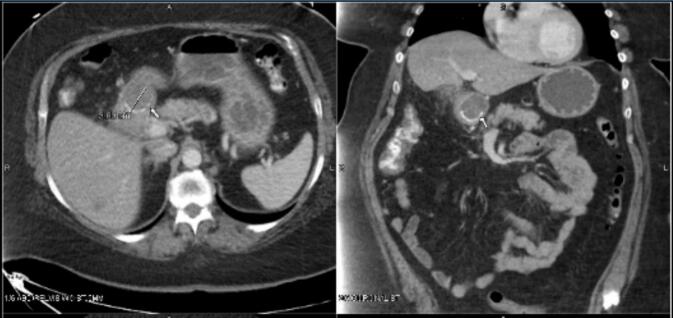
Patient 1's CT scan of the abdomen and pelvis showing large gallstone in the duodenum.

The patient's laboratory results were largely unremarkable at presentation except for a mild hypokalemia, consistent with her history of recurrent vomiting. A contrast-enhanced computed tomography (CT) scan of the abdomen and pelvis revealed a cholecystoduodenal fistula, with a 3 cm gallstone impacted in the proximal duodenum, resulting in gastric outlet obstruction (Fig. 1).

A nasogastric tube was placed for gastric decompression and the patient was taken to the operating room. An upper midline incision was made and the gallstone was palpated within the proximal duodenum and gently milked back into the stomach. A 4 cm gastrostomy was then created, allowing for the successful removal of the gallstone from the stomach. The gastrostomy was closed in two layers using absorbable sutures. The cholecystoduodenal fistula was palpable with significant surrounding inflammation and adhesions. The fistula and gallbladder were left intact.

Postoperatively, the patient experienced immediate resolution of her symptoms. She was admitted for monitoring of bowel function, with her diet gradually advanced. She was discharged in stable condition on postoperative day two. Over the subsequent two years, the patient has remained asymptomatic, reporting no episodes of abdominal pain, nausea, vomiting, or difficulty with oral intake.

### 2.2 Patient #2

**Fig. 2:**
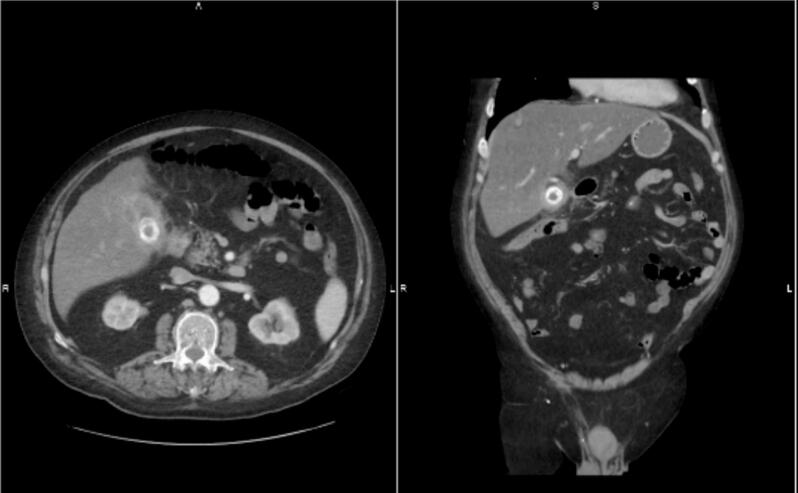
Patient 2's CT scan of the abdomen and pelvis showing a large gallstone within the gallbladder.

A 68-year-old male had multiple admissions over six months for recurrent right upper quadrant (RUQ) pain, diarrhea, and anorexia. The patient's past medical history consisted of atrial fibrillation on Pradaxa, prostate cancer, ulcerative proctocolitis, heart failure with an ejection fraction of 40 %, hypertension, and gastroesophageal reflux disease. Surgical history included robotic prostatectomy, mitral valve repair, and knee surgery. The patient's labs were significant for mildly elevated liver function tests, a total bilirubin of 1.2, and no leukocytosis. A CT abdomen/pelvis demonstrated a 3 cm gallstone within the gallbladder with significant gallbladder wall thickening and extrahepatic bile duct dilation up to 12 mm (Fig. 2). Given the patient's recent use of Pradaxa and the associated elevated risk of operative bleeding, the decision was made to manage the condition initially with a percutaneous cholecystostomy tube. The patient was discharged with the tube in place and plans for outpatient surgical follow-up.

However, the planned surgical intervention was delayed due to four hospitalizations over the subsequent six months, and the patient did not attend follow-up. The first admission was for weakness secondary to dehydration caused by *Clostridium difficile* (C. diff) infection. The second was for near-syncope and hypotension, attributed to a second episode of C. diff. The third hospitalization was due to respiratory compromise from bilateral pulmonary emboli. The fourth admission was for recurrent acute cholecystitis, necessitating the placement of a new percutaneous cholecystectomy tube.

**Fig. 3:**
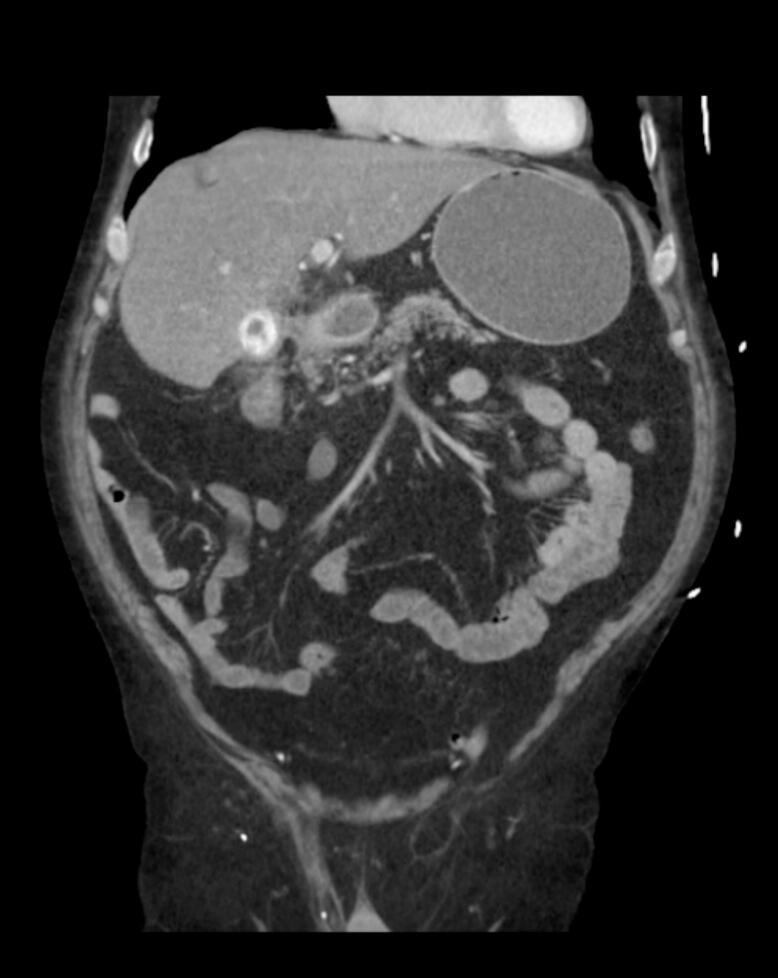
Patient 2's repeat abdominal and pelvic CT scan showing a large gallstone within the duodenum.

Approximately one month later, the patient returned to the emergency department with a one-week history of right upper quadrant abdominal pain, nausea, and vomiting. Laboratory results were unremarkable. The CT abdomen/pelvis showed a similarly inflamed gallbladder as in previous scans, but inflammation now extends to the duodenum with luminal narrowing. The stomach was noted to be dilated, which was concerning for gastric outlet obstruction. However, no fistulous tract was identified on the CT scan (Fig. 3).

The patient was stable and had recently taken Pradaxa, so it was decided to wait three days for surgery. A nasogastric tube was placed for decompression. The following day, an IVC filter was placed as the patient had a recent history of deep vein thrombosis and pulmonary embolism. On hospital day three, the patient was taken to the operating room for a diagnostic laparoscopy. Significant inflammation was found in the right upper quadrant - transverse colon, duodenum, liver, gallbladder, and omentum were adhered together. After considerable time spent dissection structures apart, the gallbladder was identified, and an abscess cavity was found near it. The abscess cavity was drained. The large stone was removed from the duodenum via the gallbladder in a piecemeal fashion and the accessible portion of the gallbladder was resected – leaving behind a sizable portion of the gallbladder due to the inflammation.

Postoperatively, the patient had significant improvement in symptoms. On post-op day three, an upper gastrointestinal (UGI) study was performed, revealing a persistent gastric outlet obstruction, likely secondary to duodenal inflammation. By post-op day eight, a CT scan showed resolution of the gastric outlet obstruction. The patient tolerated diet advancement and was discharged shortly thereafter.

This patient did well over the next two years after his surgery. He reports needing to watch his diet as fatty food can still give him some minor gastrointestinal upset and intermittent diarrhea. However, with a low-fat diet, he feels well and denies having any recurrent symptoms since his surgery.

## 3 Discussion

Endoscopic management remains the primary treatment option for Bouveret Syndrome for obstructing stones less than 4 cm in size [6]. However, there are reports that endoscopic treatment has been insufficient in patients with gallstones larger than 2.5 cm, giving conflicting evidence [4,5]. It is a highly skill-dependent, time-consuming, and challenging procedure, with previously established success rates below 10 % [7]. Due to the large size of these patients' gallstones, the risks of endoscopic removal were increased, as well as the likelihood that endoscopic management would not be successful.

Current literature recommends the removal of the stone through a gastrostomy or enterotomy in the first operation, with a possible second operation consisting of cholecystectomy and fistula repair [8]. The primary concern of not performing interval cholecystectomy and fistula repair is the possibility of recurrence of symptoms. For these patients, the first operations consisted of a gastrotomy with stone removal and gastrotomy closure and partial cholecystectomy with stone removal, respectively, without cholecystectomy or fistula closure. While two-stage surgical management is the current recommendation in young patients, meaning younger than 74, postoperative mortality rates in patients with comorbidities who undergo cholecystectomy may reach a level greater than 12 % [8]. A restrictive surgical treatment, meaning leaving the gallbladder in place without fistula repair, has been associated with lower morbidity and mortality [9]. Two years post-operation, neither patient has had abdominal pain, nausea, or oral intake issues, thus creating the question of whether a second operation with cholecystectomy and fistula closure is necessary. Lack of recurrent symptoms provides evidence that fistula closure may not be necessary and may worsen patient outcomes due to present comorbidities and the risks of undergoing a second surgery in the absence of recurrent symptoms.

## 4 Conclusion

The available case reports and data on Bouveret Syndrome are not conclusive and, in some instances, contradictory. In the two cases presented, we observed young patients—unlike the average demographic affected by Bouveret Syndrome—who achieved resolution of their symptoms simply through gallstone removal. Furthermore, these patients have not experienced recurrent symptoms in the past two years. This suggests that they either spontaneously closed their fistulas via bile preferentially draining through the biliary tree or, more likely, they developed a new bile drainage pathway that is effective enough to prevent the formation of large gallstones. It is likely that after cholecystoduodenal fistula formation, bile had a new pathway to drain from the gallbladder, preventing bile stasis and stone formation.

Based on these cases, we believe that fistula closure should be considered only in cases where patients experience a recurrence of symptoms, as the second surgery can carry significant morbidity risks. However, there is the possibility that with additional time from treatment these patients may develop recurrent symptoms or complications and a larger sample size is needed to fully understand the outcomes for these patients. This case report was completed using the SCARE checklist [10].

## Author contribution

Carrie Tackett – wrote paper.

Patrick Stahl – Wrote paper.

Anthony McCloud – wrote paper.

Joseph Eisner – research concept development, guidance and final editing.

## Consent

Written informed consent was obtained from the patient for publication of this case report and accompanying images. A copy of the written consent is available for review by the Editor-in-Chief of this journal on request.

## Ethical approval

Study determined to be exempt from ethical approval as it does not meet the department of health and human services definition of research by Community Memorial Hospital IRB of Ventura, CA on 2/5/25.

## Guarantor

Carrie Tackett.

## Research registration number

N/a.

## Funding

None.

## Conflict of interest statement

None.

## References

[1] Nuño-Guzmán C.M. (2016). Gallstone ileus, clinical presentation, diagnostic and treatment approach. World J. Gastrointestinal. Surg..

[2] Halabi W.J. (2014). Surgery for gallstone ileus: a nationwide comparison of trends and outcomes. Ann. Surg..

[3] Cappell M.S., Davis M. (2006). Characterization of Bouveret’s syndrome: a comprehensive review of 128 cases. Am. J. Gastroenterol..

[4] Haddad F.G., Mansour W., Deeb L. (2018). Bouveret's syndrome: literature review. Cureus.

[5] Ferhatoğlu M.F., Kartal A. (2020). Bouveret’s syndrome: a case-based review, clinical presentation, diagnostics and treatment approaches. Sisli Etfal Hastan Tip Bul.

[6] Dumonceau J.M., Devière J. (2016). Novel treatment options for Bouveret’s syndrome: a comprehensive review of 61 cases of successful endoscopic treatment. Expert. Rev. Gastroenterol. Hepatol..

[7] Lowe A.S. (2005). Duodenal obstruction by gallstones (Bouveret’s syndrome): a review of the literature. Endoscopy.

[8] Gan S. (2008). More than meets the eye: subtle but important CT findings in Bouveret’s syndrome. AJR Am. J. Roentgenol..

[9] Nickel F. (2013). Bouveret’s syndrome: presentation of two cases with review of the literature and development of a surgical treatment strategy. BMC Surg..

[10] Sohrabi C., Mathew G., Maria N., Kerwan A., Franchi T., Agha R.A. (2023). The SCARE 2023 guideline: updating consensus Surgical CAse REport (SCARE) guidelines. Int. J. Surg. Lond. Engl..

